# Supplementary material for: Empirical methods for controlling false positives and estimating confidence in ChIP-Seq peaks
Source: BMC Bioinformatics. 2008 Dec 5;9:523. doi: 10.1186/1471-2105-9-523 (PMC2628906; doi:10.1186/1471-2105-9-523)
Supplement: Additional File 1 — A variety of html documents from the USeq web site detailing the available applications, their best usage, output file type descriptions, command line menus, etc. [file 1471-2105-9-523-S1.zip › USeqUserGuides/JavaDoc.zip!/JavaDoc/util/apps/gump/Gump.html]

Gump


|  |  |  |
| --- | --- | --- |
| **Overview** \| **Package** \| **Class** \| **Use** \| **Tree** \| **Deprecated** \| **Index** \| **Help** | |  |
| PREV CLASS   **NEXT CLASS** | **FRAMES**    **NO FRAMES**     **All Classes** |  |
| SUMMARY: NESTED \| FIELD \| CONSTR \| METHOD | DETAIL: FIELD \| CONSTR \| METHOD |  |


---


## util.apps.gump Class Gump

```
java.lang.Object
  util.apps.gump.Gump
```

---

``` public class Gump extends java.lang.Object ```

---

| **Field Summary** | |
| --- | --- |
| `static java.util.regex.Pattern` | `flag` |
| `static java.util.regex.Pattern` | `startSpace` |


**Constructor Summary**

`Gump(java.lang.String[] args)`


| **Method Summary** | |
| --- | --- |
| `void` | `checkMenu(java.lang.String[] command)` |
| `void` | `checkMenuParsing()` |
| `java.lang.String` | `getAppDescription()` |
| `javax.swing.JTextArea` | `getAppDescriptionS()` |
| `javax.swing.JTextArea` | `getAppName()` |
| `java.lang.String` | `getDescription()` |
| `java.io.File` | `getJarFile()` |
| `java.lang.String` | `getJava()` |
| `java.lang.String[]` | `getMenu()` |
| `Option[]` | `getOptions()` |
| `static void` | `main(java.lang.String[] args)` |
| `void` | `makeSwingComponents()` |
| `void` | `parseMenu()` |
| `static void` | `printDocs()` |
| `void` | `processArgs(java.lang.String[] args)`             This method will process each argument and assign new varibles |
| `void` | `setAppDescription(java.lang.String appDescription)` |
| `void` | `setAppDescriptionS(javax.swing.JTextArea appDescriptionS)` |
| `void` | `setAppName(javax.swing.JTextArea appName)` |
| `void` | `setDescription(java.lang.String description)` |
| `void` | `setJarFile(java.io.File jarFile)` |
| `void` | `setJava(java.lang.String java)` |
| `void` | `setMenu(java.lang.String[] menu)` |
| `void` | `setOptions(Option[] parameters)` |

**Methods inherited from class java.lang.Object**

`equals, getClass, hashCode, notify, notifyAll, toString, wait, wait, wait`

**Field Detail**

### startSpace

```
public static final java.util.regex.Pattern startSpace
```

---


### flag

```
public static final java.util.regex.Pattern flag
```


**Constructor Detail**

### Gump

```
public Gump(java.lang.String[] args)
```


**Method Detail**

### makeSwingComponents

```
public void makeSwingComponents()
```

---


### checkMenuParsing

```
public void checkMenuParsing()
```

---


### checkMenu

```
public void checkMenu(java.lang.String[] command)
```

---


### parseMenu

```
public void parseMenu()
```

---


### main

```
public static void main(java.lang.String[] args)
```

---


### processArgs

```
public void processArgs(java.lang.String[] args)
```

:   This method will process each argument and assign new varibles

---


### printDocs

```
public static void printDocs()
```

---


### getJarFile

```
public java.io.File getJarFile()
```

---


### setJarFile

```
public void setJarFile(java.io.File jarFile)
```

---


### getMenu

```
public java.lang.String[] getMenu()
```

---


### setMenu

```
public void setMenu(java.lang.String[] menu)
```

---


### getDescription

```
public java.lang.String getDescription()
```

---


### setDescription

```
public void setDescription(java.lang.String description)
```

---


### getOptions

```
public Option[] getOptions()
```

---


### setOptions

```
public void setOptions(Option[] parameters)
```

---


### getJava

```
public java.lang.String getJava()
```

---


### setJava

```
public void setJava(java.lang.String java)
```

---


### getAppName

```
public javax.swing.JTextArea getAppName()
```

---


### setAppName

```
public void setAppName(javax.swing.JTextArea appName)
```

---


### getAppDescription

```
public java.lang.String getAppDescription()
```

---


### setAppDescription

```
public void setAppDescription(java.lang.String appDescription)
```

---


### getAppDescriptionS

```
public javax.swing.JTextArea getAppDescriptionS()
```

---


### setAppDescriptionS

```
public void setAppDescriptionS(javax.swing.JTextArea appDescriptionS)
```


---


|  |  |  |
| --- | --- | --- |
| **Overview** \| **Package** \| **Class** \| **Use** \| **Tree** \| **Deprecated** \| **Index** \| **Help** | |  |
| PREV CLASS   **NEXT CLASS** | **FRAMES**    **NO FRAMES**     **All Classes** |  |
| SUMMARY: NESTED \| FIELD \| CONSTR \| METHOD | DETAIL: FIELD \| CONSTR \| METHOD |  |


---
